# Diagnostic performance of biomarkers for ovarian cancer: Protocol for an overview, evidence mapping, and adjusted indirect comparisons

**DOI:** 10.1097/MD.0000000000015508

**Published:** 2019-05-03

**Authors:** Jinyong Hua, Jing Liu, Mengge Hua, Runjin Cai, Muyang Li, Jing Wang, Jiancheng Wang

**Affiliations:** a Zhengzhou Central Hospital Affiliated to Zhengzhou University, Zhengzhou; b Public People's Hospital of Xinzheng, Xinzheng; c The Second Clinical Medical College of Lanzhou University; d Department of Obstetrics and Gynecology, First Hospital of Lanzhou University; e Gansu Provincial Hospital, Lanzhou, China.

**Keywords:** adjusted indirect comparison, biomarker, diagnostic test accuracy, evidence mapping, ovarian cancer, overview

## Abstract

**Background:**

Ovarian cancer is one of the deadliest gynecological diseases and the annual mortality of ovarian cancer continues to rise. The prognosis of ovarian cancer is poor because it is prone to early metastasis during progression. Therefore, early diagnosis of ovarian cancer is very important. Some systematic reviews have evaluated the diagnostic value of different biomarkers for ovarian cancer. However, there is no consensus in the conclusions, and some are even contradictory. This study aims to assess the methodological and reporting quality of available systematic reviews and to find an optimal biomarker for diagnosing ovarian cancer.

**Methods:**

The PubMed, Embase.com, the Cochrane Library of Systematic Reviews, and Web of Science were searched to identify relevant systematic reviews from inception to February 2019. We included systematic reviews that include randomized controlled trials, cross-sectional studies, case-control studies, or cohort studies as long as the systematic reviews evaluated the diagnostic performance of biomarkers for ovarian cancer. The methodological quality will be assessed using assessment of multiple systematic reviews-2 checklist, and the reporting quality will be assessed using preferred reporting items for systematic reviews and meta-analysis diagnostic test accuracy (PRISMA-DTA) checklist. The pairwise meta-analysis and indirect comparisons will be performed using STATA (13.0; Stata Corporation, College Station, TX).

**Results:**

The results of this overview will be submitted to a peer-reviewed journal for publication.

**Conclusion:**

This overview will provide comprehensive evidence of different biomarkers for diagnosing ovarian cancer.

**PROSPERO registration number:**

CRD42019125880.

## Abstract 2

Supplemental Digital Content is available in the text

## 1 Introduction

Ovarian cancer is the seventh most common female cancer in the world,^[1]^ but it is one of the deadliest gynecological diseases, with 295,414 new cases and 184,799 death in 2018 worldwide.^[2–4]^ The incidence and annual mortality of ovarian cancer continue to rise, especially in developing countries.^[5,6]^ Although most patients can respond to the first treatment, the prognosis is still poor, as ovarian cancer is prone to early metastasis during its progression.^[7,8]^ Previous studies have shown that 5-year survival rate in patients with advanced ovarian cancer is only 30% after treatment, while 5-year survival rate in early ovarian cancer patients is as high as 92.7%.^[9–11]^ Therefore, there is an urgent need to identify some biomarkers for early diagnosis of ovarian cancer to improve its prognosis.

Fortunately, many scholars have devoted themselves to exploring potential biomarkers for diagnosing ovarian cancer during the past several years. The carbohydrate antigen 125 (CA125) was first evaluated in the early 1980s, but this marker has low sensitivity in the early stages of ovarian cancer.^[12–14]^ The study conducted by Yanaranop et al^[15,16]^ in 2017 indicated that the specificity of Human Epididymis Protein 4 (HE4) was 86%, and the AUC of HE4 (0.893) was higher than CA125 (0.865). Cao et al^[17]^ and Zuberi et al^[18]^ revealed that microRNAs may be a potential biomarker for the diagnosis and prognosis of ovarian cancer. However, which biomarker is the optimal option for diagnosing ovarian cancer remains unclear.

Well-conducted systematic reviews (SRs) and meta-analyses of randomized controlled trials (RCTs) are often considered the best way to obtain evidence of healthcare decisions.^[19–21]^ Recently, some SRs have evaluated the diagnostic value of different biomarkers for ovarian cancer.^[22–25]^ However, there is no consensus in the conclusions, and some are even contradictory. Thus, it is crucial to re-evaluate these SRs. The objectives of this overview are: to assess the methodological and reporting quality of available SRs; to evaluate diagnostic accuracy of biomarkers for ovarian cancer by reanalyzing the results of meta-analysis; to compare the diagnostic value of different biomarkers with adjusted indirect comparisons.

## 2 Methods

### 2.1 Design and registration

We will conduct an overview of SRs of diagnostic test accuracy. As a part of our project, the protocol has been registered on international prospective register of systematic review (PROSPERO) (CRD42019125880). We will follow the Preferred Reporting Items for Systematic Reviews and Meta-analysis^[26]^ statements for reporting our overview.

### 2.2 Eligibility criteria

#### 2.2.1 Type of study

We will include SRs that include randomized controlled trials, cross-sectional studies, case-control studies, or cohort studies as long as the SRs evaluated the diagnostic performance of biomarkers for ovarian cancer. The SRs should report inclusion/exclusion criteria, adequate search strategy, sufficient details about the included studies, the diagnostic value of at least 1 biomarker, the data of diagnostic value such as sensitivity, specificity, and diagnostic odds ratio (DOR).

#### 2.2.2 Type of participants

We will include ovarian cancer patients regardless of the treatment regimen and tumor staging. There are no limitations in age, race, or nationality.

#### 2.2.3 Type of interventions

Any type of biomarker is used to diagnose ovarian tumor including some common tumor biomarkers and some tumor-specific biomarkers. The index test can be one biomarker or one biomarker combines with other biomarkers.

#### 2.2.4 Type of outcomes

The primary outcomes are sensitivity, specificity, positive likelihood ratio, negative likelihood ratio, diagnostic odds ratio, area under the curve, and their respective 95% confidence intervals (CIs). The second outcomes are methodological and reporting quality of included SRs, and the relative diagnostic estimates of different biomarkers.

#### 2.2.5 Other criteria

SRs will be excluded from the overview including diagnostic tests of imaging modalities; SRs without meta-analysis; review protocols and methodological articles.

### 2.3 Search strategy

The search strategy has been developed and tested through an iterative process by an experienced medical information specialist in consultation with the review team.^[27]^ A combination of subject terms and keywords was used and make appropriate adjustments of vocabulary and grammar between different databases. The PubMed, Embase.com, the Cochrane Library of Systematic Reviews, and Web of Science were searched to identify relevant SRs from inception to February 2019. There was no restriction on the language of publication. In addition, the reference lists of included SRs have been checked for additional references. The search strategy of PubMed can be found in Supplementary 1.

### 2.4 Study selection

The literature search records will be imported into EndNote X8 (Thomson Reuters [Scientific] LLC Philadelphia, PA) literature management software. After removing duplicates, 2 independent reviewers will examine the title and abstract of studies found in the search to identify related studies. Then, the same 2 reviewers will retrieve the full text of all possibly relevant studies and assess the eligibility of each study according to the eligibility criteria. Conflicts will be resolved by a third reviewer.

### 2.5 Data extraction

To detect and resolve overlapping SRs, we will first map the research questions and characteristics of all eligible SRs. If we identify multiple reviews addressing the same research question that are eligible for inclusion but share the same primary study, we will use the following standard hierarchy to select a review to include in the overview: the review with the highest methodological quality rating; the most recent review; the review with the larger number of studies included.^[28]^ We will extract study characteristics from SRs including the following items: author name, year of publication, country of corresponding author, number of author, journal name, country of journal, funding, disease, number and name of biomarkers, number and name of reference test, and outcomes; methodological characteristics of SRs such as types of included studies, number of included studies, samples, number and name of databases retrieved, and supplemental literature search; results of statistical analysis including pooled sensitivity, specificity, likelihood ratio, predictive value, diagnostic odds ratio, area under curve, and their 95% CI. Full data abstraction will be completed by 1 reviewer and verified by a second reviewer. Disagreements will be resolved by consensus or by discussion with a third reviewer.

### 2.6 Quality assessment

The Assessment of Multiple Systematic Reviews (AMSTAR), published in 2007, consists of 11 items. Previous studies found that the AMSTAR is a reliable methodological quality assessment tool with good agreement, construct validity, and feasibility.^[29–31]^ But it was developed to evaluate SRs of randomized trials. AMSTAR-2, a major revision of the original AMSTAR instrument, could be used to assess SRs based on non-RCTs.^[32,33]^ Thus, we will use it to assess the methodological quality of included SRs.

The preferred reporting items for systematic reviews and meta-analysis diagnostic test accuracy (PRISMA-DTA), consists of 27 items, is an expanded checklist of original PRISMA, which aims to improve the completeness and transparency of reporting of SRs of diagnostic test accuracy studies.^[34]^ We will use it to assess the reporting quality of included SRs. Two review authors will independently assess the risk of bias in each study according to predefined criteria. Disagreements regarding by-item and overall rating of quality will be resolved by consensus or third-party adjudication if consensus cannot be reached.

### 2.7 Data synthesis

#### 2.7.1 Evidence map

Map the biomarkers. We will create a bubble plot according to the biomarkers for all included SRs. This map displays information in 3 dimensions the bubble size represents the total number of reviews, the total number of participants included in the SRs in the x-axis, the biomarkers in the *y*-axis. Map the quality. The bubble plot will be produced according to the methodological and the reporting quality, where each bubble represents 1 SR. The information of 3 dimensions in the map is the bubble size represents the number of primary studies included in the SRs, the methodological quality in the *x*-axis, the reporting quality in the *y*-axis.

#### 2.7.2 Pairwise meta-analysis

We will perform a pairwise meta-analysis with the data of pooled sensitivity, specificity, DOR, positive likelihood ratio, negative likelihood ratio and their 95% CI lower limit, 95% CI upper limit using Mantel-Haenszel statistical method with STATA (13.0; Stata Corporation, College Station, TX). The heterogeneity between each study will be estimated using the *P* value and the inconsistency index (*I*^2^ test). If the *I*^2^ is ≤50%, it suggests that there is negligible statistical heterogeneity, and the fixed effects model will be employed. If the *I*^2^ is >50%, we will explore sources of heterogeneity by subgroup analysis and meta-regression. If there is no clinical heterogeneity, the random effects model will be used to perform the meta-analysis. Otherwise, clinical heterogeneity will be explored through discussion with the review team.

#### 2.7.3 Adjusted indirect comparisons

We will calculate relative diagnostic outcomes between index tests including relative sensitivity, relative specificity, and relative DOR. Then, we will conduct indirect comparisons using relative diagnostic outcomes.

#### 2.7.4 Assessment of publication bias

Publication bias will be assessed per biomarker; therefore, if we have >10 SRs evaluated the same biomarker then the evidence of funnel plot asymmetry will be assessed using the Begg test using a *P* value of 0.1 to acknowledge the low power of this test.^[28]^

#### 2.7.5 Subgroup analysis

If sufficient data are available, we will perform subgroup analysis on the basis of the age, body mass index, and ethnicity of participants; the country in which the study was conducted; the cutoff and time period of biomarkers; the quality of the SRs.

## 3 Discussion

This study will identify all relevant SRs that reported the diagnostic value of biomarkers for ovarian cancer. In addition to assessing the methodological and reporting quality of included SRs, we will also reanalyze the results of the meta-analysis using a pairwise meta-analysis and an adjusted indirect comparison. What is more, we will present the biomarkers and quality using the bubble plot, which can clearly show the biomarkers and quality of each SR. We hope this overview will find an excellent biomarker for diagnosing ovarian cancer and the results can help clinicians and patients choose an optimal diagnostic method for detecting ovarian cancer.

## Author contributions

Jinyong Hua, Jing Wang, and Jiancheng Wang planed and designed the research. Jing Liu, Mengge Hua, Runjin Cai, and Muyang Li tested the feasibility of the study. Jing Wang and Jiancheng Wang provided methodological advice, polished and revised the manuscript. Jinyong Hua and Jiancheng Wang wrote the manuscript. All authors approved the final version of the manuscript.

**Conceptualization:** Jinyong Hua.

**Data curation:** Jing Liu, Mengge Hua, Muyang Li.

**Funding acquisition:** Jiancheng Wang.

**Investigation:** Jinyong Hua, Jing Liu, Mengge Hua, Runjin Cai, Muyang Li.

**Methodology:** Jing Wang, Jiancheng Wang.

**Project administration:** Jinyong Hua, Jing Wang.

**Resources:** Jing Liu, Runjin Cai, Jing Wang.

**Supervision:** Jiancheng Wang.

**Validation:** Jiancheng Wang.

**Writing – original draft:** Jinyong Hua, Jing Wang.

**Writing – review & editing:** Jing Wang, Jiancheng Wang.

## Supplementary Material

Supplemental Digital Content

## References

[1] Coburn SB Bray F Sherman ME International patterns and trends in ovarian cancer incidence, overall and by histologic subtype. Int J Cancer 2017;140:2451–60. 28257597 10.1002/ijc.30676 PMC5595147

[2] Bray F Ferlay J Soerjomataram I Global cancer statistics 2018: GLOBOCAN estimates of incidence and mortality worldwide for 36 cancers in 185 countries. CA Cancer J Clin 2018;68:394–424. 30207593 10.3322/caac.21492

[3] Lowe KA Chia VM Taylor A An international assessment of ovarian cancer incidence and mortality. Gynecol Oncol 2013;130:107–14. 23558050 10.1016/j.ygyno.2013.03.026

[4] Matulonis UA Sood AK Fallowfield L Ovarian cancer. Nat Rev Dis Primers 2016;2:16061. 27558151 10.1038/nrdp.2016.61 PMC7290868

[5] Chen W Zheng R Baade PD Cancer statistics in China, 2015. CA Cancer J Clin 2016;66:115–32. 26808342 10.3322/caac.21338

[6] Huang Z Zheng Y Wen W Incidence and mortality of gynecological cancers: secular trends in urban Shanghai, China over 40 years. Eur J Cancer 2016;63:1–0. 27254837 10.1016/j.ejca.2016.04.016 PMC4942399

[7] Fields MM Chevlen E Ovarian cancer screening: a look at the evidence. Clin J Oncol Nurs 2006;10:77–81. 16482731 10.1188/06.CJON.77-81

[8] Wang X Kong D Wang C Circulating microRNAs as novel potential diagnostic biomarkers for ovarian cancer: a systematic review and updated meta-analysis. J Ovarian Res 2019;12:24. 30898156 10.1186/s13048-019-0482-8 PMC6427862

[9] Tew WP Ovarian cancer in the older woman. J Geriatr Oncol 2016;7:354–61. 27499341 10.1016/j.jgo.2016.07.008

[10] van Jaarsveld MT Helleman J Boersma AW miR-141 regulates KEAP1 and modulates cisplatin sensitivity in ovarian cancer cells. Oncogene 2013;32:4284–93. 23045278 10.1038/onc.2012.433

[11] Beavis AL Smith AJ Fader AN Lifestyle changes and the risk of developing endometrial and ovarian cancers: opportunities for prevention and management. Int J Women's Health 2016;8:151–67. 27284267 10.2147/IJWH.S88367 PMC4883806

[12] Bast RC Feeney M Lazarus H Reactivity of a monoclonal antibody with human ovarian carcinoma. J Clin Invest 1981;68:1331–7. 7028788 10.1172/JCI110380 PMC370929

[13] Urban N McIntosh MW Andersen M Ovarian cancer screening. Hematol Oncol Clin North Am 2003;17:989–1005. 12959188 10.1016/s0889-8588(03)00063-7

[14] Dochez V Caillon H Vaucel E Biomarkers and algorithms for diagnosis of ovarian cancer: CA125, HE4, RMI and ROMA, a review. J Ovarian Res 2019;12:28. 30917847 10.1186/s13048-019-0503-7 PMC6436208

[15] Yanaranop M Anakrat V Siricharoenthai S Is the risk of ovarian malignancy algorithm better than other tests for predicting ovarian malignancy in women with pelvic masses? Gynecol Obstet Investig 2017;82:47–53. 27197526 10.1159/000446238

[16] Wilailak S Chan KK Chen CA Distinguishing benign from malignant pelvic mass utilizing an algorithm with HE4, menopausal status, and ultrasound findings. J Gynecol Oncol 2015;26:46–53. 25310857 10.3802/jgo.2015.26.1.46 PMC4302285

[17] Cao Q Lu K Dai S Clinicopathological and prognostic implications of the miR-200 family in patients with epithelial ovarian cancer. Int J Clin Exp Pathol 2014;7:2392–401. 24966949 PMC4069884

[18] Zuberi M Mir R Das J Expression of serummiR-200a, miR-200b, and miR-200c as candidate biomarkers in epithelial ovarian cancer and their association with clinicopathological features. Clin Transl Oncol 2015;17:779–87. 26063644 10.1007/s12094-015-1303-1

[19] Ge L Tian JH Li YN Association between prospective registration and overall reporting and methodological quality of systematic reviews: a meta-epidemiological study. J Clin Epidemiol 2018;93:45–55. 29111471 10.1016/j.jclinepi.2017.10.012

[20] Tian JH Zhang J Ge L The methodological and reporting quality of systematic reviews from China and the USA are similar. J Clin Epidemiol 2017;85:50–8. 28063911 10.1016/j.jclinepi.2016.12.004

[21] Yao L Sun R Chen YL The quality of evidence in Chinese meta-analyses needs to be improved. J Clin Epidemiol 2016;74:73–9. 26780259 10.1016/j.jclinepi.2016.01.003

[22] Fakhar HB Rezaie-Tavirani M Zali H Comparison of serum human epididymis protein (HE4), carbohydrate antigen 125 (CA125) and risk of ovarian malignancy algorithm (ROMA) as markers in ovarian cancer: a systematic review and a meta-analysis. Ind J Gynecol Oncol 2018;16:10.

[23] Zhen S Bian LH Chang LL Comparison of serum human epididymis protein 4 and carbohydrate antigen 125 as markers in ovarian cancer: a meta-analysis. Mol Clin Oncol 2014;2:559–66. 24940495 10.3892/mco.2014.279 PMC4051564

[24] Dayyani F Uhlig S Colson B Diagnostic performance of risk of ovarian malignancy algorithm against CA125 and HE4 in connection with ovarian cancer: a meta-analysis. Int J Gynecol Cancer 2016;26:1586–93. 27540691 10.1097/IGC.0000000000000804

[25] Zuo S Yang G Hu F Combined detection of CA125, CA19-9, and CEA in the diagnosis of ovarian cancer: a meta analysis. Chin J Clin Oncol 2012;39:263–8.

[26] Moher D Liberati A Tetzlaff J Preferred reporting items for systematic reviews and meta-analysis: the PRISMA statement. Int J Surg 2010;8:336–41. 20171303 10.1016/j.ijsu.2010.02.007

[27] Li L Tian J Tian H Network meta-analyses could be improved by searching more sources and by involving a librarian. J Clin Epidemiol 2014;67:1001–7. 24841794 10.1016/j.jclinepi.2014.04.003

[28] Fordham B Sugavanam T Hopewell S Effectiveness of cognitive-behavioural therapy: a protocol for an overview of systematic reviews and meta-analyses. BMJ Open 2018;8:e025761. 10.1136/bmjopen-2018-025761 PMC6303684 30552285

[29] Shea BJ Grimshaw JM Wells GA Development of AMSTAR: a measurement tool to assess the methodological quality of systematic reviews. BMC Med Res Methodol 2007;7:10. 17302989 10.1186/1471-2288-7-10 PMC1810543

[30] Shea BJ Hamel C Wells GA AMSTAR is a reliable and valid measurement tool to assess the methodological quality of systematic reviews. J Clin Epidemiol 2009;62:1013–20. 19230606 10.1016/j.jclinepi.2008.10.009

[31] Li XX Zheng Y Chen YL The reporting characteristics and methodological quality of Cochrane reviews about health policy research. Health Policy 2015;119:503–10. 25260911 10.1016/j.healthpol.2014.09.002

[32] Yan P Yao L Li H The methodological quality of robotic surgical meta-analyses needed to be improved: a cross-sectional study. J Clin Epidemiol 2019;109:20–9. 30579979 10.1016/j.jclinepi.2018.12.013

[33] Shea BJ Reeves BC Wells G AMSTAR 2: a critical appraisal tool for systematic reviews that include randomised or non-randomised studies of healthcare interventions, or both. BMJ 2017;358:j4008. 28935701 10.1136/bmj.j4008 PMC5833365

[34] McInnes MDF Moher D Thombs BD Preferred reporting items for a systematic review and meta-analysis of diagnostic test accuracy studies: the PRISMA-DTA Statement. JAMA 2018;319:388–96. 29362800 10.1001/jama.2017.19163

